# Understanding Community Perspectives on *Staphylococcus aureus* Disease and Prevention in the White Mountain Apache Tribal Community in Arizona

**DOI:** 10.3390/ijerph23070845

**Published:** 2026-06-27

**Authors:** Sumayya U. Beekun, Monica Pilewskie, Catherine G. Sutcliffe, Francene Larzelere Sinquah, Shea J. Littlepage, Jennifer R. Richards, Natalie Jones, Laura L. Hammitt

**Affiliations:** Center for Indigenous Health, Department of International Health, Johns Hopkins Bloomberg School of Public Health, Baltimore, MD 21205, USA; sbeekun1@alumni.jh.edu (S.U.B.); mpilews1@alumni.jh.edu (M.P.); csutcli1@jhu.edu (C.G.S.); flarzel1@jhu.edu (F.L.S.); shea.littlepage@jhu.edu (S.J.L.); jricha81@jhu.edu (J.R.R.); njones65@jhu.edu (N.J.)

**Keywords:** *Staphylococcus aureus*, Indigenous health, infection prevention, community-based research, White Mountain Apache, MRSA, culturally tailored interventions

## Abstract

**Highlights:**

**Public health relevance—How does this work relate to a public health issue?**

- Indigenous communities experience disproportionately high *Staphylococcus aureus* infection burden not addressed by standard prevention strategies.
- This study examines community perceptions of infection causes, stigma, and care-seeking within an Indigenous community and Tribal healthcare setting to inform and improve intervention design.

**Public health significance—Why is this work of significance to public health?**

- Acceptability of prevention strategies depends on cultural fit, trusted messengers, and feasibility rather than biomedical efficacy alone.
- Findings will be used to inform educational materials to strengthen health literacy and the design of a clinical trial aimed at reducing Staph carriage.

**Public health implications—What are the key implications or messages for practitioners, policy makers and/or researchers in public health?**

- Effective infection prevention should combine biomedical strategies with culturally grounded education and community partnership.
- Community-engaged formative research can provide transferable context and support for designing sustainable interventions with Indigenous and other underserved communities.

**Abstract:**

*Staphylococcus aureus* (Staph) infections are a pressing health concern in the White Mountain Apache (WMA) Tribal community, where invasive Staph infection rates far exceed those in the general U.S. population. This study explored community perspectives to guide culturally tailored education and prevention strategies. We conducted 42 in-depth interviews and focus group discussions with healthcare providers, traditional practitioners, and community members. Thematic analysis showed that participants had familiarity with the term “MRSA” (methicillin-resistant Staph), although many did not recognize it as a form of Staph, per se. Barriers to timely care-seeking included lack of transportation, stigma, and misconceptions about infection causes. With regard to biomedical approaches to prevention, participants preferred products like antiseptic nasal sprays and antimicrobial skin cleansers due to ease of use. Community members emphasized the need for simple, bilingual educational materials grounded in Apache culture and delivered by trusted figures. The findings underscore the importance of culturally grounded education and prevention approaches. Implementation and scaling of these strategies may enhance health literacy, reduce infection rates, and promote holistic wellness in Indigenous communities.

## 1. Introduction

*Staphylococcus aureus* (Staph) is a bacterium that commonly colonizes the skin and nasal passages of healthy individuals but can also cause serious infections, including skin and soft tissue infections (SSTIs), sepsis, and pneumonia [1]. Of particular concern is methicillin-resistant *S. aureus* (MRSA), which is resistant to standard antibiotic treatments and associated with substantial morbidity and mortality [2,3,4]. The burden of Staph infections is not equally distributed across populations; Indigenous communities in the United States face significantly higher rates of disease than their non-Indigenous counterparts [3,5,6,7,8,9,10]. Recent studies from the Navajo Nation and White Mountain Apache Tribal lands confirm disproportionately high incidence of invasive MRSA and methicillin-sensitive *S. aureus* compared with the general U.S. population [6,7].

This disparity is particularly pronounced in the White Mountain Apache (WMA) Tribal community, where the incidence of invasive MRSA infections has been reported to be more than seven times higher than in the general U.S. population [6,11]. The community also has a high burden of skin infections, with rates of hospitalization for skin and soft tissue infections, more than half of which were associated with Staph, 20 times the general U.S. population [8]. Social and structural determinants of health—high household occupancy, limited access to clean water, and transportation barriers—exacerbate the risk of infection and delay access to timely care [10,11]. These inequities are compounded by historical and ongoing experiences of colonization, medical mistrust, and underinvestment in public health infrastructure [10,12,13,14,15,16].

While previous research has documented the epidemiology of Staph in Indigenous communities, fewer studies have explored community perceptions, preferences, and lived experiences that shape infection prevention and care-seeking behaviors. There is growing recognition that effective public health interventions must be culturally grounded, community-informed, and co-developed with Indigenous partners to achieve health equity [10,12,13,14,16,17,18,19,20,21,22]. Community-based research models, such as Indigenous Community-Based Participatory Research (ICBPR), promote self-determination and ensure that interventions reflect local values, languages, and knowledge systems [13,16,20,21,22,23,24,25,26,27,28,29]. ICBPR often promotes community input and guidance through a community advisory board (CAB), composed of community and cultural stakeholders. Community-engaged approaches are increasingly recognized as essential for addressing persistent health inequities in Indigenous communities because they support Indigenous leadership, cultural relevance, and sustainable intervention development.

This study addresses a critical gap by applying qualitative methods to understand how WMA community members, traditional practitioners, and healthcare providers conceptualize Staph infections and to evaluate the feasibility and acceptability of prevention strategies. In this study, prevention approaches were broadly conceptualized to include biomedical products with demonstrated efficacy in reducing the presence of Staph as well as community-driven education. Through in-depth interviews (IDI) and focus group discussions (FGD), we aimed to surface culturally relevant insights that would guide the development of educational materials and interventions to reduce Staph carriage and infections.

## 2. Materials and Methods

**Figure 1:**
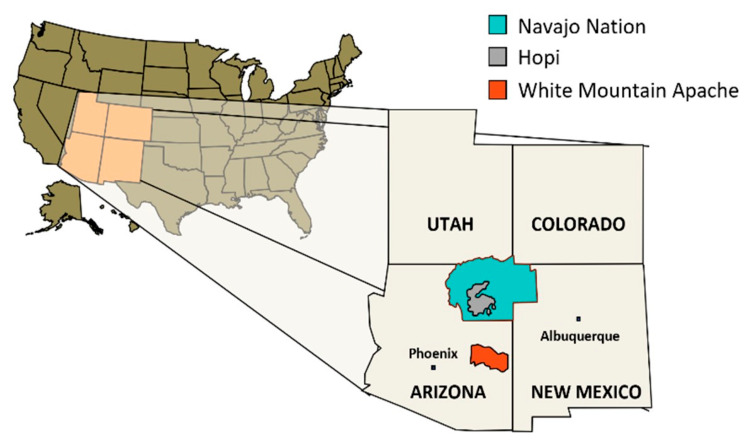
Map of the White Mountain Apache Tribal lands (orange), located in the southwestern United States.

This qualitative study was conducted in the WMA Tribal community in eastern Arizona (See Figure 1), a population that has experienced a high burden of invasive Staph infections [6,30]. There are over 12,000 WMA tribal members residing on 1.6 million acres on the Fort Apache Indian Reservation [30]. The study was developed in partnership with a WMA Community Advisory Board and Indian Health Service (IHS) providers, using a community-engaged research approach.

In this research, we are centered on Indigenous perspectives. There are many terms used to describe the Indigenous Peoples of the United States, such as Native American, American Indian, and Alaska Native. For this article, the term ‘Indigenous’ was used to describe this community. Decolonization is a clinical term that refers to a biomedical protocol using antimicrobials, such as intranasal mupirocin and antiseptic body washes, to reduce the presence of Staph. In this research, we refer to biomedical approaches as Staph prevention strategies because the term decolonization in Indigenous Research generally refers to a “process which engages with imperialism and colonialism” and “privileges the indigenous presence… and that acknowledges our continuing existence” [13].

### 2.1. Study Design and Recruitment

We used purposive sampling to recruit a diverse set of participants, including healthcare providers, community members, and traditional practitioners. Eligibility criteria for community participants included self-identifying as an Indigenous adult (18 years or older) and living in or near the WMA Tribal lands. Traditional practitioners were recruited with the assistance of Tribal partners, while healthcare providers were selected based on their roles in treating SSTIs or patients at increased risk of Staph infection at the IHS Hospital serving tribal members.

### 2.2. Data Collection

Data were collected between June and October 2023. Use of IDIs allowed participants to discuss personal experiences, healthcare encounters, and sensitive topics such as stigma in a private setting, while FGDs facilitated discussion of shared community perspectives, norms, and recommendations for prevention strategies. Together, these approaches provided complementary perspectives and enhanced the breadth of data collected.

The interview guides were developed collaboratively with a WMA Community Advisory Board and the research team, drawing on prior literature on Staph, community health priorities identified by the Community Advisory Board, and the research questions guiding this study. The guide followed a semi-structured format, which ensured consistent exploration of key domains (e.g., knowledge of Staph infections, experiences with care-seeking, and preferences for prevention strategies) while allowing participants flexibility to share perspectives in their own words.

Trained Indigenous interviewers conducted individual IDIs and FGDs using the semi-structured interview guides tailored to each group. Interviews were conducted in English. One IDI was conducted virtually, and the rest were in person. IDIs took 60–90 min, and FGDs took 90–120 min. Discussions focused on community knowledge of Staph, prevention strategies, perceived barriers to care, and preferences for educational materials and prevention products. During IDIs and FGDs, potential prevention products were described and demonstrated, and participants were asked to rank them in order of preference (1 = most preferred; 6 = least preferred). Prevention products included antiseptic skin cleansers (e.g., Hibiclens) in the form of a wash or cloth to be used several times a week; antiseptic nasal sprays (e.g., Nozin^®^ nasal sanitizer) to be used twice per day; a topical antibiotic ointment to be used daily for 5 days in the nose; bleach baths to be used 2–3 times per week for up to a month; and a hypothetical oral probiotic pill used daily. Rankings were compiled and analyzed descriptively to identify relative preferences across participants.

### 2.3. Data Analysis

All sessions were audio-recorded, transcribed verbatim, and uploaded to Dedoose U.S. Version 9.0.107 qualitative analysis software [31]. An inductive thematic analysis approach was used to code transcripts [32,33]. A coding team of two researchers (SUB and MP) reviewed transcripts and developed an initial codebook. Intercoder reliability was established through independent double-coding of 20% of transcripts [34].

Findings from IDIs and FGDs were analyzed together. Similarities and differences across participant groups and data collection methods were examined during team discussions and incorporated into theme development. Emerging themes were refined through iterative team discussions and consensus-building. When culturally specific concepts emerged during discussions, excerpts were reviewed with the Community Advisory Board to support interpretation and ensure the cultural relevance of emerging themes.

### 2.4. Ethics

The study protocol was approved by the WMA Tribal Council and the Johns Hopkins Bloomberg School of Public Health and Indian Health Service Phoenix Area Institutional Review Boards. All participants provided written informed consent before participation.

## 3. Results

A total of 42 participants (26 female and 16 male) were enrolled in the study, including healthcare providers (*n* = 17), community members (*n* = 23), and traditional practitioners (*n* = 2). The median age was 47 years (interquartile range [IQR]: 38, 66) for community members and 45 years (IQR: 35, 54) for healthcare providers. Healthcare providers from the following nine departments participated: Preventive Medicine, Emergency, Family Care Unit, Diabetes Clinic, Wound Clinic, Pharmacy, Obstetrics, Pediatrics, and Outpatient Clinic. The following provider roles were represented: physician, nurse, physical therapist, medical assistant, and pharmacist. All healthcare providers and traditional practitioners, as well as 10 community members, participated in IDIs. Three FGDs were conducted with 13 community member participants.

From participants’ responses, nine key themes were identified through thematic analysis and are presented below with supporting quotes and interpretations. Table 1 also provides a summary of the nine themes and key finding(s) of each theme.

### 3.1. High Community Recognition of MRSA/Low Recognition of Staph Infections

Participants demonstrated high awareness of the term “MRSA” and its risks, but many did not associate MRSA with *S. aureus* or recognize the term “Staph.” Community members frequently attributed skin infections to factors such as bug bites or skin irritation. Despite this, they actively sought information during clinic appointments and expressed eagerness to learn. As one participant shared, “I just realized that Staph is something serious after hearing from the doctor.” Another participant remarked on the confusion between terms: “People know about the MRSA part, but if you call it a Staph infection, they don’t know what that is.” These insights highlight an opportunity and a desire for improving health literacy.

### 3.2. Barriers to and Community Strategies for Accessing Timely Care

Community members described using family support systems, informal networks with healthcare staff, and local transportation services to overcome logistical barriers. Although stigma and long wait times sometimes discouraged care-seeking, participants showed persistence and self-advocacy. For instance, one said, “I waited one day… and then two days later it didn’t look right so I had to go.” Healthcare providers, especially nurses, were seen as knowledgeable sources of support: “Our outpatient nurses are real, like they’re the ones that help us.”

### 3.3. Preferences for Prevention Strategies and Products

Participants favored prevention products that felt familiar and easy to use, such as antiseptic washes or nasal sprays. Bleach baths were widely rejected, with participants associating them with household cleaning and noting practical barriers such as lack of bathtub access in some homes. From qualitative discussion, antiseptic washes (mean rank = 2) and antiseptic nasal sprays (mean rank = 3) or antiseptic wipes (mean rank = 3) were most preferred, while bleach baths (mean rank = 5) were least preferred. Topical antibiotic ointments had a mean rank of 4. A hypothetical oral probiotic for Staph prevention (mean rank = 2) was generally viewed favorably and received a similar ranking to the antiseptic washes when discussed. One community member noted, “I just don’t think the kids will want something done with their nose every day… some adults are stubborn about having stuff done with their nose every day,” underscoring an important consideration.

### 3.4. Barriers to and Strengthening of Health Literacy and Infection Prevention

Participants consistently described widespread misconceptions about Staph, often attributing infections to bug bites, allergies, or other unrelated causes. These misunderstandings contributed to delays in seeking care and confusion about effective prevention strategies. Limited access to accurate health information and inconsistent messaging from different sources further compounded these knowledge gaps. Participants emphasized that educational interventions must address these barriers directly by clearly explaining how Staph is transmitted, how it differs from other skin conditions, and why prevention matters.

Community members expressed a strong desire for “simple messaging… in both Apache and English… so people can really understand it” and highlighted the importance of trusted messengers, such as community health workers and nurses, in delivering this information. Participants also pointed to structural barriers—such as limited access to hygiene resources and the scarcity of culturally relevant materials—as factors that undermined prevention efforts. Addressing these challenges was viewed as a crucial step in strengthening infection prevention and empowering community members to make informed health decisions.

### 3.5. Integrating Traditional and Community-Based Healing Approaches

Traditional healing was viewed as a vital complement to biomedical care. Herbal remedies, ceremonies, and spiritual healing were respected approaches to infection management. Participants emphasized the need for collaboration between traditional and clinical providers to foster trust and cultural resonance: “In Apache way, we had medicines before IHS hospital.”

### 3.6. Building Community Support and Reducing Stigma

Stigma was cited as a major barrier to care, particularly when infections were visible or linked to head lice or skin conditions. Participants called for normalization of discussions about Staph through community-wide education (e.g., radio, Apache Scout newsletter, tabling at grocery store, and brochures at the IHS clinic waiting area and in local housing authority materials shared with residents). One healthcare provider noted, “There shouldn’t be any shame in that,” referring to skin infections in children.

### 3.7. Enhancing Information Access and Reducing Misinformation

Participants often relied on Facebook, family stories, and local networks for information—sources that may perpetuate misinformation. This reliance reflected both a gap in accessible, medically accurate resources and a need for trustworthy, culturally relevant education. One participant explained, “Sometimes you just gotta go on intuition… if you can’t find it, sometimes the nurses are very helpful.”

### 3.8. Developing Community-Centered, Standardized Healthcare Protocols

Healthcare providers called for consistent treatment protocols, especially for recurring infections. Providers expressed a desire for protocols that were both standardized and responsive to local needs and practices. One provider suggested, “We could have a more standardized approach of offering decolonization to people with like two or more infections in six months.”

### 3.9. Leveraging Community Strengths for Health Promotion

Participants demonstrated resilience, resourcefulness, and a desire to improve health outcomes through collective action. They did this by advocating for family members, pushing for community-wide education, and proposing interventions rooted in cultural practice and values. These included integrating prevention education into early childhood home visiting programs, senior centers, and family-oriented spaces, as well as creating materials targeting caregivers, peers, and family members.

## 4. Discussion

This formative qualitative study explored WMA community members’ and healthcare providers’ perceptions, experiences, and preferences related to Staph infections and prevention strategies. The findings revealed a complex landscape of strengths, barriers, and opportunities that can guide the design of interventions tailored to Indigenous communities and local contexts. Across interviews and focus groups, participants described strong motivation to learn, cultural ways for understanding health, and a collective willingness to engage in prevention—while also highlighting persistent structural barriers, knowledge gaps, and systemic limitations.

Participants demonstrated strong awareness of MRSA, though the connection to Staph more broadly was often unclear. Infections were attributed to non-infectious causes such as insect bites or allergies, underscoring a gap in understanding the etiology of Staph infections. Like other studies, as well as materials from the WHO and CDC [12,14,15,20,21,22,23,24,28,29], we found that these misconceptions contributed to delays in care-seeking and confusion about prevention strategies, highlighting a critical need for educational tools that clearly explain transmission, risk, and prevention in accessible, culturally resonant language. Community members repeatedly expressed a desire for “simple messaging… in both Apache and English” and emphasized the importance of trusted messengers—such as nurses, community health workers, and local leaders—in delivering accurate information [13,16,17,25].

Despite knowledge gaps, participants actively sought information and demonstrated resilience and self-advocacy when navigating barriers to care. Transportation challenges, long wait times, and limited clinic availability were common obstacles, yet community members leveraged family networks, peer support, and personal initiative to overcome them [15,20,22,28]. Nurses were repeatedly cited as critical sources of both clinical guidance and emotional support, often serving as the first point of contact for concerns about skin infections and offering culturally sensitive explanations about treatment and prevention. This reliance on nursing staff underscores their central role in bridging biomedical care and community understanding.

Preferences for prevention strategies reflected both cultural acceptability and practical considerations. Participants favored antiseptic washes, nasal sprays, and topical treatments that were familiar, easy to use, and feasible within their daily routines, while bleach baths were widely rejected due to associations with household cleaning and concerns about access to bathing facilities. These preferences and logistical challenges need to be considered when recommending or prescribing prevention strategies to increase uptake and adherence. Similarly, participants valued educational approaches that were visual, bilingual, and embedded within existing community communication channels—such as radio, newsletters, and family-based education—rather than delivered solely in clinical settings [14,28].

The study also illuminated how prevention was conceptualized not only as a biomedical task but also as a cultural and community-driven responsibility. Traditional healing practices—including herbal remedies, ceremonies, and spiritual healing—were viewed as essential complements to biomedical care. Participants emphasized the importance of collaboration between IHS providers and traditional practitioners to optimally care for patients. Recent policy shifts in Arizona, such as the inclusion of traditional healers as billable providers within the Arizona Health Care Cost Containment System (i.e., Medicaid), may further support such collaborations by opening new pathways to integrate traditional healing services into broader healthcare systems.

Reducing stigma emerged as another central component of effective prevention [21,26]. Participants noted that visible infections were often associated with shame, leading to delays in care. Normalizing conversations about skin health—through community-wide education, trusted messengers, and accessible resources—was seen as a critical step toward improving early treatment and reducing disease burden. Participants also described reliance on informal information networks, including social media and family stories, which sometimes perpetuated misinformation. This finding highlights the need for proactive, credible, and culturally relevant health communication strategies to counter myths and build trust [14,15,20,24,28].

Participants’ recommendations extended beyond traditional health education, calling for interventions embedded in community life. Such approaches not only expand the reach of health promotion but also reinforce prevention as a shared responsibility across generations [12,13,27].

Importantly, participants underscored that prevention strategies must be feasible within the context of local resources and infrastructure. Interventions that combine biomedical efficacy with cultural resonance—such as pairing antiseptic treatments with traditional practices, delivering education through trusted figures, and aligning messaging with Apache language and values—are likely to be more acceptable, sustainable, and impactful than interventions focused solely on biomedical approaches [13]. The call by providers for standardized clinical protocols, particularly for recurring infections, also reflects a desire for consistency and trustworthiness in care, aligning with national recommendations for culturally adapted infection prevention guidelines in Indigenous communities [14,15,18,20,23,25,28,35].

This study has several limitations. First, findings reflect perspectives from a single Indigenous community and, while they may be similar to other communities with similar socio-economic determinants, they are not generalizable to all Indigenous communities. Second, participants were recruited using purposive sampling, which may not capture the full diversity of experiences within the White Mountain Apache community. Third, responses may have been influenced by social desirability bias. Despite these limitations, the study provides important formative insights grounded in community perspectives and priorities. Taken together, these findings provide critical insights for designing interventions that are both medically effective and culturally congruent. They emphasize the importance of centering Indigenous leadership, integrating traditional knowledge with biomedical care, and embedding prevention within the social fabric of the community. By leveraging community strengths—such as collective action, advocacy, and resilience—and addressing structural barriers and misinformation, Staph prevention strategies can become not only more effective but also more empowering and equitable.

Future research should evaluate the effectiveness, acceptability, and implementation of culturally grounded Staph prevention interventions developed from these findings. Additional studies across diverse Indigenous communities may identify both shared and community-specific approaches to infection prevention.

## 5. Conclusions

This study provides formative insights into how the WMA community understands, experiences, and responds to Staph infections. Participants exhibited strong community engagement, a desire for culturally grounded education, and resilience in navigating structural barriers. Preferences for accessible and familiar prevention products, integration of traditional knowledge, and support for bilingual, visual educational materials highlight a clear path forward for culturally responsive prevention strategies.

The findings emphasize the value of community-driven approaches in designing interventions that honor Indigenous perspectives, strengthen trust, and empower communities to lead health promotion efforts. These lessons offer an approach for adapting infection prevention strategies in other Indigenous communities, where similar challenges—and strengths—may exist.

Future implementation efforts should continue to center Indigenous leadership, community collaboration, build on existing community assets, and ensure that Staph prevention tools are both medically effective and culturally congruent.

## Figures and Tables

**Table 1:** Summary table of themes identified from in-depth interviews and focus group discussions.

Theme	Key Finding(s)
High Recognition of MRSA, Low Recognition of Staph	The term “MRSA” was more familiar than “Staph”
Accessing Timely Care	Transportation and clinic wait times were barriers
Prevention Preferences	Antiseptic washes and nasal sprays were preferred
Health Literacy	Misconceptions about causes of infection
Traditional Healing	Traditional and biomedical approaches viewed as complementary
Reducing Stigma	Shame delayed care-seeking
Information Access	Reliance on social networks
Standardized Protocols	Providers desired consistency
Community Strengths	Community resilience and advocacy

## Data Availability

Data collected in the White Mountain Apache Tribal lands are owned by the Tribe. The data presented in this study can be made available upon request to the corresponding author (contact lhammitt@jhu.edu), if consistent with the Institutional Review Board-approved protocol and if the disclosure is approved by the Tribe.

## Abbreviations

The following abbreviations are used in this manuscript:

FGD	Focus Group Discussion
IDI	In-depth interview
IHS	Indian Health Service
NARCH	Native American Research Centers for Health
WMA	White Mountain Apache Tribe
